# Spinal metaplasticity in respiratory motor control

**DOI:** 10.3389/fncir.2015.00002

**Published:** 2015-02-11

**Authors:** Daryl P. Fields, Gordon S. Mitchell

**Affiliations:** Department of Comparative Biosciences, University of Wisconsin-MadisonMadison, WI, USA

**Keywords:** respiratory control, respiratory plasticity, metaplasticity, spinal cord, motor neuron, intermittent hypoxia, phrenic motor neuron

## Abstract

A hallmark feature of the neural system controlling breathing is its ability to exhibit plasticity. Less appreciated is the ability to exhibit metaplasticity, a change in the capacity to express plasticity (i.e., “plastic plasticity”). Recent advances in our understanding of cellular mechanisms giving rise to respiratory motor plasticity lay the groundwork for (ongoing) investigations of metaplasticity. This detailed understanding of respiratory metaplasticity will be essential as we harness metaplasticity to restore breathing capacity in clinical disorders that compromise breathing, such as cervical spinal injury, motor neuron disease and other neuromuscular diseases. In this brief review, we discuss key examples of metaplasticity in respiratory motor control, and our current understanding of mechanisms giving rise to spinal plasticity and metaplasticity in phrenic motor output; particularly after pre-conditioning with intermittent hypoxia. Progress in this area has led to the realization that similar mechanisms are operative in other spinal motor networks, including those governing limb movement. Further, these mechanisms can be harnessed to restore respiratory and non-respiratory motor function after spinal injury.

## Introduction

As with most neural systems, a hallmark of the neural system controlling breathing is its ability to express plasticity; defined as a persistent (>60 min) change in neural network function after an experience/stimulus has ended (Mitchell and Johnson, 2003). Respiratory plasticity is characteristic of development, continues throughout life, and is of considerable importance in preserving life when confronted with clinical disorders that compromise the ability to breathe, including diseases of the lung and chest wall, or neurological disorders such as spinal injury and motor neuron disease (Vinit et al., 2009; Dale-Nagle et al., 2010; Nichols et al., 2013; Dale et al., 2014).

Less well known is that respiratory plasticity itself adapts based on experience (Mitchell and Johnson, 2003), a phenomenon referred to as metaplasticity or “plastic plasticity” (Abraham and Bear, 1996). Although there are examples of respiratory plasticity and metaplasticity in sensory receptors, including the carotid body (CB) chemoreceptors (Kumar and Prabhakar, 2012); prominent examples of respiratory plasticity and metaplasticity are found at the other end of the respiratory control system, the motor nuclei (Mahamed and Mitchell, 2007; Dale-Nagle et al., 2010; Devinney et al., 2013).

In this brief review, we focus on known examples of spinal respiratory motor plasticity and metaplasticity, as well as implications for clinical application. We first define respiratory plasticity, metaplasticity and related concepts, and then the potential sites of respiratory plasticity, models of spinal respiratory plasticity/metaplasticity, possible mechanisms of metaplasticity, and finally, gaps in our knowledge that require additional research.

## Definitions: (meta) plasticity and (meta) modulation

Modulation and plasticity are related but distinct properties that are frequently confused. Higher order properties such as metamodulation and metaplasticity are less frequently considered and even more often confused. Thus, brief definitions of these terms are provided in the context of respiratory motor control (Mitchell and Johnson, 2003); although they also apply to limb motor control (reviewed in Grau et al., 2014).

*Modulation* is a change in system behavior that fades rapidly (seconds to minutes) after the stimulus is removed. Neuromodulators often work through metabotropic G protein coupled receptors, which alter cell excitability through covalent modifications of membrane channels. Modulation does confer system flexibility and can initiate cellular mechanisms resulting in plasticity (Mitchell and Johnson, 2003), but the two are differentiated by what happens when the stimulating trigger is removed. For example, during a brief (5 to 30 min) hypoxic experience, phrenic nerve activity (and breathing) increases and returns to normal seconds/minutes after hypoxia has ceased. Alternatively, 3 successive 5-min hypoxic episodes give rise to a persistent increase in phrenic nerve activity lasting several hours after the final hypoxic episode has ended—an expression of plasticity (see below). In this example, modulation is the within episode (during hypoxia) augmentation of respiratory motor output, plasticity is the persistence of increased activity that lasts long after the hypoxia stimulus has ended.

*Meta-modulation* is a reversible change in the capacity or quality of modulation (Katz and Edwards, 1999; Mitchell and Johnson, 2003), and requires continued presence of the meta-modulation trigger. Meta-modulating stimuli also frequently act through G protein coupled receptors, 2^nd^ messengers and/or ion channels to augment the response of neurons to modulators (Katz and Edwards, 1999; Mesce, 2002; Ribeiro and Sebastiao, 2010), though metamodulation triggers have not yet been associated with long-term changes in gene expression. One interesting example of meta-modulation in respiratory control is the response of neurons in the nucleus of the solitary tract to concurrent serotonin and substance P application (Jacquin et al., 1989). Both serotonin and substance P alone positively modulate nucleus tractus solitarius (NTS) neurons. However, in the presence of substance P, serotonin becomes inhibitory (Jacquin et al., 1989). Thus, in these *in vitro* conditions, the impact of one modulator (serotonin) is augmented by concurrent application of another (substance P).

*Plasticity (long-term)* is a persistent (>60 min) change in function that outlasts the initiating stimulus (Mitchell and Johnson, 2003). Plasticity often requires new protein synthesis via translational and/or transcriptional regulation (Manahan-Vaughan et al., 2000; Mitchell and Johnson, 2003; Alberini, 2008), though it is not a prerequisite. A frequent initiating stimulus in many neural systems is neuronal activity, or activity-dependent synaptic plasticity (Malinow and Malenka, 2002; Wiegert and Bading, 2011); though activity-dependent plasticity is not characteristic of respiratory motor control (Mitchell and Johnson, 2003; Strey et al., 2013). Instead, neuromodulators frequently elicit respiratory plasticity through distinct signaling cascades induced by patterned metabotropic receptor activation.

One prominent model of plasticity in spinal respiratory motor control is phrenic long-term facilitation (pLTF), a long-lasting increase in phrenic motor output observed following acute intermittent hypoxia (AIH; Feldman et al., 2003; Mahamed and Mitchell, 2007; Dale-Nagle et al., 2010; Devinney et al., 2013). AIH elicits episodic serotonin release within the phrenic motor nucleus (Kinkead et al., 2001), activation of spinal serotonin receptors (Fuller et al., 2001; Baker-Herman and Mitchell, 2002) and a long-lasting enhancement of phrenic motor output (Mahamed and Mitchell, 2007; Devinney et al., 2013). Since this form of phrenic motor plasticity is initiated by intermittent, but not sustained hypoxia of similar cumulative duration, it is pattern sensitive (Baker and Mitchell, 2000; Devinney et al., 2013), similar to other forms of serotonin dependent plasticity (Sherff and Carew, 2002; Philips et al., 2013). In summary, modulatory experiences in themselves are not sufficient for plasticity as their effects fade once the trigger is removed. Alternatively, pattern specific modulation can be encoded through discrete signaling pathways to elicit a long-lasting augmentation of nerve output that persists after the triggering experience; i.e., plasticity/metaplasticity.

*Metaplasticity* is a change in the capacity for neuroplasticity (Abraham and Bear, 1996; Byrne, 1997). An important distinction between metaplasticity and metamodulation is that metaplasticity is expressed after triggering experiences (i.e., hypoxia) for both plasticity and metaplasticity are gone (Abraham, 2008). As in plasticity, metaplasticity often encodes previous experiences by altering gene expression (Mitchell and Johnson, 2003); therefore changing the ability of a system to respond to subsequent experiences. Through encoded memory, the effects of metaplasticity triggers can persists long after the stimulation has subsided; the focus of this review is to review how intermittent hypoxia training (a metaplasticity trigger) can augment subsequent spinal respiratory plasticity.

## Potential sites of respiratory plasticity and metaplasticity

Essential processes in control of breathing include respiratory rhythm generation, burst pattern formation (Feldman and Smith, 1989; Mitchell et al., 1990) as well as sensory feedback giving rise to chemoreflexes, mechanoreflexes, neuromodulation and neuroplasticity (Feldman et al., 2003; Mitchell and Johnson, 2003).

Respiratory plasticity and metaplasticity can occur in any component of the neural system controlling breathing (Mitchell and Johnson, 2003). Of particular interest to this review, plasticity and metaplasticity occur in spinal respiratory motor neurons (and/or interneurons) putting the final “touches” on burst pattern formation before the central nervous system (CNS) relays its command to breathe to respiratory muscles. Thus, plasticity and metaplasticity are important in sculpting motor output to individual respiratory muscles. Here, we focus on spinal respiratory motor plasticity, with less consideration given to plasticity in chemoreceptor feedback (Bisgard, 2000; Kumar and Prabhakar, 2012) or brainstem mechanisms of respiratory rhythm generation/pattern formation (Blitz and Ramirez, 2002; Feldman et al., 2003, 2005). We instead refer to several well-written reviews of carotid (Kumar and Prabhakar, 2012) and brainstem (Ramirez et al., 2012) plasticity, which can elicit upstream respiratory plasticity that is phenotypically similar to spinal respiratory plasticity.

**Figure 1:**
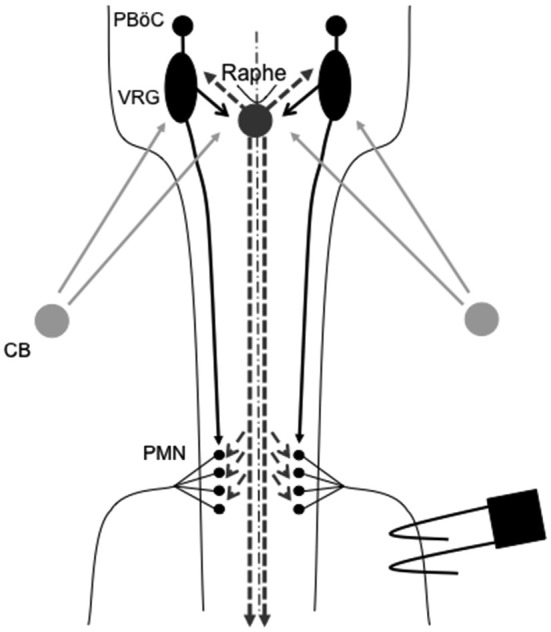
**Representation of brainstem and spinal cord regions critical for respiratory motor control**. Respiratory rhythm generation requires a small region of the medulla known as the pre-Bötzinger complex (PBöC). The central rhythm is transmitted to brainstem respiratory pre-motor neurons of the ventral respiratory group (VRG). VRG pre-motor neurons subsequently relay respiratory drive projections to different respiratory motor neuron pools, including phrenic motor neurons (PMN). Sensory input to the respiratory system during episodic hypoxia is provided by the carotid body (CB) chemoreceptors in the neck, which project via chemoafferent neurons to the medullary nucleus of the solitary tract (not shown). These second order sensory neurons subsequently project (directly or indirectly) to multiple structures of importance in ventilatory control, including the PBöC, VRG and serotonergic neurons in the medullary raphe (raphe). Raphe serotonergic neurons play a key role in phrenic long-term facilitation (pLTF) following acute intermittent hypoxia (AIH) and, presumably, in metaplasticity of pLTF.

One prominent source of modulatory input giving rise to plasticity originates from brainstem serotonergic raphe neurons that project to spinal and brainstem respiratory neurons. In particular, brainstem raphe neurons are known to modulate respiratory motor neurons and, under the right circumstances, initiate important forms of respiratory motor plasticity (Mitchell et al., 2001; Feldman et al., 2003). Figure 1 outlines the neural network controlling breathing and identifies potential sites of respiratory neuroplasticity; all of which could also exhibit metaplasticity.

## Phrenic long-term facilitation is a form of spinal respiratory motor plasticity

The most extensively studied model of spinal respiratory motor plasticity is AIH induced pLTF; a persistent increase in phrenic nerve output following 3 × 5 min experiences of moderate hypoxia (35–45 mmHg PaO2; Hayashi et al., 1993; Bach and Mitchell, 1996; Mitchell et al., 2001; Feldman et al., 2003; Mahamed and Mitchell, 2007; Dale-Nagle et al., 2010; Devinney et al., 2013). In recent years we have come to use a generic term for long-lasting enhancement of phrenic motor output; phrenic motor facilitation (pMF). Whereas pMF may arise from a variety of triggers (i.e., inactivity, hypercapnia, pharmacology), pLTF is a specific form of pMF elicited from AIH (Devinney et al., 2013).

In many instances, spinal signaling pathways that are necessary for pLTF are also independently sufficient to elicit pMF (reviewed by Dale-Nagle et al., 2010). For example, serotonin receptor antagonists applied to the C3–C5 cervical spine region during AIH abolishes pLTF; demonstrating that spinal serotonin receptor activation in the immediate vicinity of the phrenic motor nucleus is necessary for pLTF (Fuller et al., 2001; Baker-Herman and Mitchell, 2002). AIH-induced pLTF is also abolished by cervical spinal pre-treatment with siRNAs targeting BDNF mRNA (Baker-Herman et al., 2004) and neuronal nitric oxide synthase (nNOS; MacFarlane et al., 2014). Conversely, activation of cervical spinal serotonin type 2A, 2B and 7 receptors (MacFarlane and Mitchell, 2009; Hoffman and Mitchell, 2011; MacFarlane et al., 2011), TrkB receptors (the high affinity brain derived neurotrophic factor (BDNF) receptor; Baker-Herman et al., 2004), and nNOS (MacFarlane et al., 2014) can all give rise to phenotypically similar pMF in the absence of hypoxia (i.e., mimicking pLTF). Collectively, these experiments reveal important aspects of the signaling pathway giving rise to AIH-induced pLTF, but also identify important signaling checkpoints that are independently sufficient for pMF.

Though similar spinal signaling pathways can drive pLTF and other forms of pMF, it is important to differentiate between the two experimentally and conceptually (Dale-Nagle et al., 2010). For example, intrathecal drug application allows for the investigation of pMF mechanisms confined to the region of the phrenic motor nucleus. Conversely, moderate AIH-induced pLTF has global effects that influence other respiratory control sites (Figure 1) as well as non-respiratory motor pools within the thoracic and lumbar spinal segments. AIH-induced pLTF begins via hypoxia activation of peripheral (caotid body) chemoreceptors (Hayashi et al., 1993; Bach and Mitchell, 1996; Bavis and Mitchell, 2003), second order medullary neurons in the nucleus of the solitary tract, and then (indirectly) serotonergic neurons of the medullary raphe nuclei (Morris et al., 1996; Li et al., 1999; Mitchell et al., 2001; Figure 1). Subsequent episodic serotonin release on or near spinal respiratory motor neurons (Kinkead et al., 2001) initiates cellular cascades giving rise to moderate AIH (35–45 mmHg PaO2) induced pLTF (Fuller et al., 2001).

When elicited by more severe hypoxic episodes (25–30 mmHg PaO2) but otherwise the same AIH protocol (Nichols et al., 2012), AIH elicits pLTF through an entirely different mechanism; requiring spinal adenosine type 2A (A2A) receptor activation (Golder et al., 2008; Nichols et al., 2012). Our working model is that severe hypoxia triggers greater adenosine triphosphate (ATP) release from CNS cells, increasing ATP and adenosine concentrations. Elevated adenosine near phrenic motor neurons (PMN) activates A2A receptors and drives adenosine-dependent (serotonin independent) pLTF (Nichols et al., 2012). We have not yet identified the specific cellular source of adenosine for severe AIH induced pLTF. However, astrocytes increase their release of ATP and adenosine during hypoxia, and are a likely source for the relevant adenosine for severe AIH-induced pLTF (Kulik et al., 2010). Additional research is needed to confirm this hypothesis.

Although serotonin- and adenosine-dependent pLTF are phenotypically similar, they operate through distinct signaling pathways referred respectively as the Q and S pathways to pMF (Dale-Nagle et al., 2010); these pathways are named for the G proteins most often coupled to their initiating receptors (i.e., Gq for serotonin type 2 and Gs for adenosine 2A). Additionally, pMF can be induced by activation of Gq-coupled alpha-1 adrenergic receptors (Huxtable et al., 2014) or Gs-coupled 5-HT7 receptors (Hoffman and Mitchell, 2013). However, neither alpha-1 or 5-HT7 receptors are necessary for any form of AIH-induced pLTF (Hoffman and Mitchell, 2013; Huxtable et al., 2014). Thus, these receptors are sufficient for pMF, but not necessary for pLTF; they elicit pMF by convergent downstream signaling onto Q and S pathways.

The Q pathway to pMF consists of: spinal Gq-linked G protein coupled receptors activation (Fuller et al., 2001; Baker-Herman and Mitchell, 2002; MacFarlane et al., 2011; Huxtable et al., 2014), protein kinase C (PKC) activation (Devinney and Mitchell, unpublished observations), new synthesis of BDNF (Baker-Herman and Mitchell, 2002; Baker-Herman et al., 2004), TrkB receptors (Baker-Herman et al., 2004; Dale et al., unpublished), and downstream signaling via ERK MAP kinases (Wilkerson and Mitchell, 2009; Hoffman et al., 2012). In contrast, the S pathway to pMF involves: spinal Gs-linked G protein coupled receptors (Hoffman and Mitchell, 2011; Nichols et al., 2012), adenylyl cyclase activation with synthesis of cyclic AMP, new protein synthesis of an immature TrkB isoform (vs. BDNF; Golder et al., 2008; Hoffman and Mitchell, 2011) and downstream signaling via Akt (vs. ERK; Golder et al., 2008; Hoffman et al., 2012).

The Q and S pathways interact in interesting and complex ways that we have defined as “cross-talk inhibition” (Dale-Nagle et al., 2010; Hoffman et al., 2010; Hoffman and Mitchell, 2013). Our working hypothesis is that manipulations of these cross-talk interactions during intermittent hypoxia training may underlie at least some forms of spinal respiratory metaplasticity (see below). During moderate AIH-induced pLTF, serotonin release activates abundant, high affinity 5-HT2 receptors; driving the dominant Q pathway to pLTF. Although concurrent activation of Gs protein linked A2A or 5-HT7 receptors is insufficient to trigger the S pathway at these levels of hypoxia (moderate AIH-induced pLTF is exclusively Q pathway dependent plasticity), sub-threshold activation of these Gs-linked receptors constrains the Q pathway and reduces pLTF magnitude. Inhibition of spinal A2A receptors (Hoffman et al., 2010) and/or 5-HT7 receptors (Hoffman and Mitchell, 2013) during moderate AIH eliminates this cross-talk constraint thereby enhancing moderate AIH-induced pLTF. We suspect that all Gs-linked G protein coupled receptors have a capacity to constrain the Q pathway to pMF since cross-talk inhibition is mediated by protein kinase A (PKA); a prominent effector of downstream Gs protein/cAMP signaling. Whereas spinal PKA inhibition relieves cross-talk inhibition and enhances moderate AIH induced pLTF (to a similar extent as A2A and 5-HT7 inhibition), PKA activation suppresses moderate AIH-induced pLTF (Hoffman and Mitchell, 2013). Since Gs-linked G protein coupled receptors are commonly expressed within many CNS cell types, we cannot yet state that cross-talk inhibition is operative within a single neuron; spinal motor neurons. It remains possible that elements of the Q and S pathways, and their interactions, arise from interneuron, astrocyte and/or microglia. The cellular localization of processes involved in spinal respiratory plasticity is an area that requires and warrants additional research.

Independent from the contributions of G protein coupled receptors, spinal activation of JAK2-coupled erythropoietin (EPO; Dale et al., 2012) or tyrosine kinase coupled vascular endothelial growth factor (VEGF; Dale-Nagle et al., 2011) receptors is sufficient to elicit pMF. EPO, VEGF, and their high affinity receptors are all oxygen-regulated proteins with increased expression following prolonged hypoxia (Semenza et al., 1991; Forsythe et al., 1996). We suspect that these proteins play a relatively greater role in phrenic motor plasticity during conditions of prolonged hypoxia experiences (days-months). Both VEGF- and EPO-induced pMF are Akt and ERK dependent, suggesting downstream divergence onto both the Q and S signaling pathways (Dale-Nagle et al., 2011; Dale et al., 2012). The diversity of hypoxia-induced mechanisms giving rise to phenotypically similar pMF most likely reflects the need to adapt to hypoxia that differs in quality (severity, pattern) and duration. Specifically, we suggest that differential responses with severity (i.e., Q to S pathways) and duration (serotonin/adenosine to VEGF/EPO; Dale et al., 2014) enable unique interactions for adaptive flexibility during hypoxia. Further, this diversity of responses may enable emergent plasticity properties, such as metaplasticity.

## pLTF Metaplasticity induced by intermittent hypoxia preconditioning

### Chronic intermittent hypoxia (CIH)

**Figure 2:**
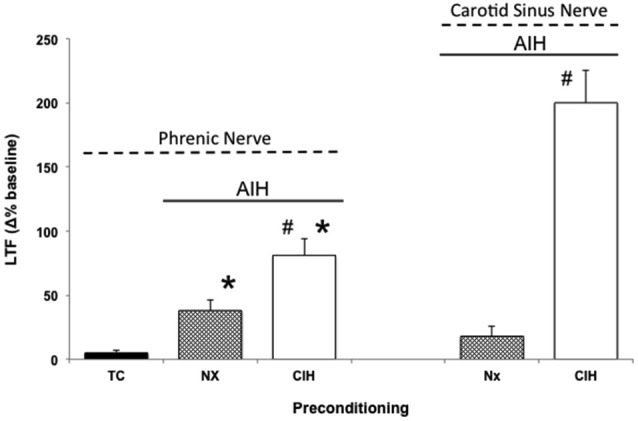
**Metaplasticity in AIH induced pLTF and carotid (chemo)sensory long-term facilitation (csLTF) following preconditioning with chronic intermittent hypoxia (CIH)**. On left, time controls (TC) are without AIH, and no pLTF is observed. In contrast, AIH elicits pLTF in rats pretreated with both normoxia (Nx) and CIH (*p < 0.05 vs. TC). However, AIH-induced pLTF is greater in rats preconditioned with CIH vs. Nx (^#^*p* < 0.05 vs. Nx), demonstrating meta-plasticity in pLTF (data from Ling et al., 2001). On right, sensory recordings of the carotid sinus nerve reveal no csLTF in rats exposed to AIH following Nx preconditioning (post 60 min). However, after CIH preconditioning, robust csLTF is observed, revealing a form of plasticity not present in normal rats (i.e., metaplasticity; data from Peng and Prabhakar, 2003).

CIH in rodents simulates some aspects of the intermittent hypoxia experienced during obstructive sleep apnea (OSA) in humans (Lee et al., 2009). Thus, when rodents are preconditioned with CIH, pathogenesis ensues (Reeves and Gozal, 2006; Moraes et al., 2012; Prabhakar and Semenza, 2012; Navarette-Opazo and Mitchell, 2014), yet multiple distinct forms of respiratory plasticity can also be observed. These include: (1) increased baseline breathing (Ling et al., 2001); (2) increased baseline carotid sinus chemoafferent neuron activity (Peng and Prabhakar, 2003, 2004); (3) decreased synaptic strength from carotid chemoafferent neurons onto their NTS targets (Kline et al., 2007); and (4) increased short-term hypoxic phrenic response (Ling et al., 2001; Peng and Prabhakar, 2003, 2004). In addition, CIH also triggers metaplasticity, expressed as enhanced AIH-induced pLTF (Ling et al., 2001), increased tidal volume in response to AIH within unanesthetized rats (i.e., ventilatory LTF; McGuire et al., 2003, 2004), and sensory LTF of carotid chemoafferents neurons (sensory LTF); a form of plasticity observed only after CIH preconditioning (Figure 2; Peng and Prabhakar, 2003, 2004). In normal rodents, AIH does not elicit sensory LTF; after CIH pretreatment, robust sensory LTF is observed after the same AIH stimulus (i.e., persistent enhancement of carotid chemoafferent activity; Peng and Prabhakar, 2003, 2004). Although the newly revealed sensory LTF may contribute to the CIH-enhanced AIH-induced pLTF, considerable evidence suggests that this is not the entire story.

Enhanced pLTF could occur and/or be modified at any site in the neural network transmitting chemosensory activity to PMN; including chemoreceptors themselves, their afferent terminations in the nucleus of the solitary tract, neurons of the ventral respiratory column, raphe neurons, spinal phrenic interneurons and/or PMN (Figure 1). There is currently no published evidence that CIH-induced pLTF metaplasticity results from changes in brainstem modulatory neurons, although prolonged CIH (four, but not one week; Bach and Mitchell, unpublished observations) increases serotonergic terminal density in the phrenic motor nucleus; suggesting that anatomical plasticity of medullary raphe neuron projections may contribute to enhanced pLTF (McCrimmon et al., 1995; Kinkead et al., 1998). Further evidence for CIH-induced CNS plasticity (vs. at the peripheral chemoreceptors) and pLTF metaplasticity includes observations that CIH: (1) increases phrenic nerve responses to electrical activation of the cut, central end of the carotid sinus nerve, bypassing carotid chemoreceptors *per se* (Ling et al., 2001); (2) attenuates synaptic input into the nucleus of the solitary tract (i.e., wrong way to account for enahnced pLTF; Kline et al., 2007, 2009); and (3) strengthens spinal pathways to PMN following cervical injury (Fuller et al., 2003).

Although relatively little is known concerning sites and signaling mechanisms of CIH metaplasticity, CIH-enhanced moderate AIH-induced pLTF is still serotonin-dependent (Ling et al., 2001; McGuire et al., 2004). On the other hand, it is no longer dependent on 5-HT2 receptors alone (Ling et al., 2001; McGuire et al., 2004). Rather, the inhibitory actions of 5-HT7 receptors on moderate AIH-induced pLTF seen in normal rats (Hoffman and Mitchell, 2013) convert to excitatory actions; both 5-HT7 and 5-HT2 receptors now contribute to enhanced LTF in CIH trained rats (McGuire et al., 2004). Thus, CIH may disrupt normal cross-talk interactions between the Q and S pathways without compromising the respective Q and S contributions to pMF (enabling additive contributions). Mechanisms accounting for this apparent loss of cross-talk inhibition between 5-HT2 and 5-HT7 receptors are not known.

Because CIH elicits respiratory metaplasticity, we initially had contemplated CIH as a treatment option to restore respiratory control in clinical disorders that impair breathing capacity; such as cervical spinal injury (Fuller et al., 2003; Mitchell, 2007). Unfortunately, CIH has well-documented adverse consequences in multiple physiological systems; including systemic hypertension (Fletcher et al., 1992), metabolic syndrome (Li et al., 2005; Tasali and Ip, 2008) and hippocampal cell death with associated cognitive deficits (Row, 2007; Row et al., 2007). Thus, although CIH may have beneficial effects in restoring respiratory control in clinical disorders that compromise breathing, undesirable side effects limit its utility. An interesting corollary is that the increased sleep apnea prevalence in individuals with chronic spinal injuries (Sankari et al., 2014) may represent a form of “self medication,” inducing spontaneous functional recovery. However, other factors associated with sleep apnea such as sleep fragmentation and systemic inflammation may over-ride any benefits (McGuire et al., 2008; Hakim et al., 2012; Zhang et al., 2012; Huxtable et al., 2013).

### Repetitive acute intermittent hypoxia (rAIH)

**Figure 3:**
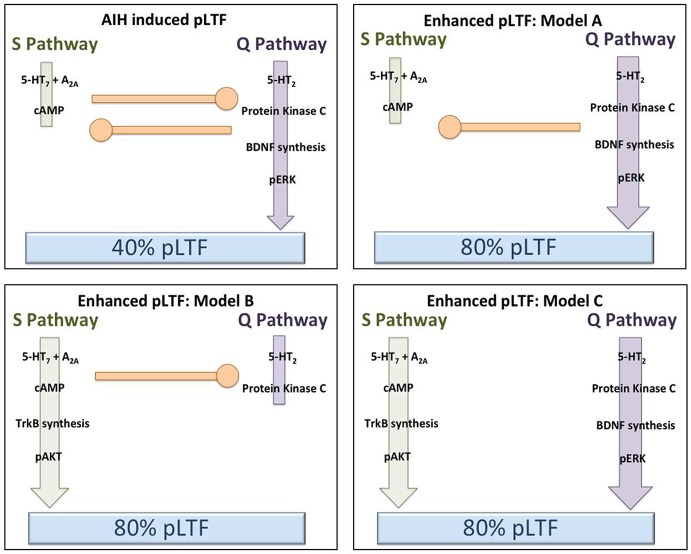
**Hypothetical models to explain pLTF metaplasticity (enhanced pLTF) after repetitive acute intermittent hypoxia (rAIH) preconditioning**. In the upper left panel, our working model of Q and S pathway contributions to pLTF in normal rats is depicted (no preconditioning). Moderate AIH normally elicits ~40% pLTF, largely via dominant contributions from the Q pathway, with concurrent restraint from sub-threshold S pathway activation (i.e., cross-talk inhibition). In the remaining panels, rAIH preconditioning enhances AIH-induced pLTF, reaching ~80% facilitation. In the upper right (Model A), we illustrate the possibility that rAIH preconditioning enhances pLTF by enhancing the Q pathway to pLTF. This could be achieved by amplifying the Q pathway, or by removing inhibition from the S pathway (while leaving Q to S inhibition intact). For example, pLTF is doubled in normal rats when cross-talk inhibition from the S pathway is reduced from cervical spinal inhibition of A2A receptors, 5-HT7 receptors or PKA (see text for references). In either case, the Q pathway remains the dominant pathway to pLTF in this scenario, similar to enhanced pLTF following chronic cervical dorsal rhizotomy (CDR) or during end-stage amyotrophic lateral sclerosis (ALS) (see text for references). In the lower left panel (Model B), we illustrate the possibility that pLTF following rAIH preconditioning arises from a reversal to dominant S pathway contributions to pLTF vs. Q pathway-dependent pLTF found in normal rats. There is little available evidence to support this possibility; however, the precedent is provided by the greater S pathway-dependent pLTF resulting from severe AIH protocols (Nichols et al., 2012). In the lower right panel (Model C), we illustrate the possibility that rAIH preconditioning somehow eliminates cross-talk inhibition and uncouples the Q and S pathways to pMF; thus, each pathway is now able to contribute to an enhanced pLTF. This possibility is supported by available evidence concerning mechanisms of enhanced pLTF after CIH pre-conditioning, where both 5-HT2 and 5-HT7 receptors appear to make independent contributions (see text).

Less intensive rAIH preconditioning protocols retain the ability to elicit plasticity and metaplasticity without the known pathogenic effects of CIH (Lovett-Barr et al., 2012; Satriotomo et al., 2012; Navarette-Opazo and Mitchell, 2014). For example, daily acute intermittent hypoxia (dAIH; 10, 5 min hypoxic episodes for seven consecutive days) increases the strength of crossed-spinal synaptic pathways to PMN (Lovett-Barr et al., 2012) and enhances moderate AIH-induced LTF (Wilkerson and Mitchell, 2009). Similarly, AIH three times per week (3xwAIH; 10, 5 min hypoxic episodes, three times per week for four or 10 weeks) increases the expression of pro-plasticity molecules within PMN (Satriotomo et al., 2012), and enhances moderate AIH-induced pLTF (MacFarlane et al., 2010; Vinit et al., 2010). In contrast to CIH, neither dAIH (Wilkerson and Mitchell, 2009; Lovett-Barr et al., 2012) or 3xwAIH (Satriotomo et al., 2012) elicit detectable systemic hypertension, hippocampal cell death or reactive gliosis. With similar “low dose” intermittent hypoxia protocols, most reported systemic effects are in fact beneficial vs. pathogenic (Dale et al., 2014; Navarette-Opazo and Mitchell, 2014). Thus, rAIH may be a simple, safe and effective means of promoting motor plasticity and functional recovery of breathing (and non-respiratory motor) deficits caused by severe clinical disorders. For example, dAIH beginning one week after cervical spinal hemisection in rats promotes remarkable functional recovery of breathing capacity (Lovett-Barr et al., 2012). Even more startling, dAIH in combination with ladder walking induced prolonged restoration of forelimb function in rats (Lovett-Barr et al., 2012). The functional benefits of intermittent hypoxia therapy may be most notable when paired with task specific training. For example, dAIH for five days increased walking endurance by 18% in humans with chronic (>nine months), incomplete spinal injuries, but increased walking endurance 38% when combined with walking practice. Conversely, walking practice alone did not improve walking endurance (Hayes et al., 2014). Although detailed mechanisms underlying rAIH induced respiratory and limb functional recovery after spinal injury are not yet known, increased expression of molecules known to be associated with both the Q and S pathways suggests that both signaling pathways may be involved (Wilkerson and Mitchell, 2009; Lovett-Barr et al., 2012; Satriotomo et al., 2012; Dale et al., 2014). Furthermore, with transcriptome analysis of gene arrays from ventral cervical segments encompassing the phrenic motor nucleus, the most significant changes noted were associated with anti-inflammatory activities. Specifically, there was a pronounced reduction in activity of the pro-inflammatory transcription factors NfκB and Stat 1/2 (Small et al., 2014).There is little direct evidence to date concerning mechanisms of rAIH-induced pLTF metaplasticity; but these studies have provided several promising leads. Additional mechanistic studies are critical to understand this highly novel and clinically translatable phenomenon. Figure 3 outlines several possible changes in inter-pathway interactions that may enhance pLTF following rAIH training. A greater understanding of rAIH-induced metaplasticity will be important as we develop rAIH into a therapeutic tool to treat motor deficits caused by spinal injury (Lovett-Barr et al., 2012; Trumbower et al., 2012; Hayes et al., 2014) or motor neuron disease (Mitchell, 2007; Nichols et al., 2013).

## Other models of pLTF metaplasticity?

AIH-induced pLTF is modified by a number of other pre-treatments that enhance and diminish its expression (Mitchell et al., 2001). However, since it is not always possible to reverse the initiating factor/treatment (i.e., metaplasticity trigger), it is sometimes ambiguous if the change in pLTF reflects true metaplasticity vs. a persistent change in the initiating stimulus. Conditions altering AIH-induced pLTF that we suspect reflect models of metaplasticity include: (1) chronic cervical dorsal rhizotomy (CDR; Kinkead et al., 1998); (2) systemic inflammation (Vinit et al., 2011; Huxtable et al., 2013); (3) cervical spinal injury in a time-dependent manner (Golder and Mitchell, 2005); (4) ALS (Nichols and Mitchell, 2014); (5) age (Zabka et al., 2001); and (6) sex hormones (Zabka et al., 2006).

Of these, the best characterized is enhanced pLTF following CDR. Twenty-eight days following bilateral CDR, moderate AIH induced pLTF is doubled, with an equivalent increase in serotonin terminal density in the immediate vicinity of identified PMN (Kinkead et al., 1998). Increased serotonin terminal density is associated with enhanced pLTF in several models of pLTF metaplasticity (see above, McCrimmon et al., 1995; Kinkead et al., 1998; Mitchell et al., 2001; Satriotomo et al., 2006). Both normal and CDR-enhanced pLTF are abolished by the 5-HT2 receptor antagonist, ketanserin, demonstrating that CDR-enhanced pLTF arises from accentuation of normal Q pathway dependent pLTF (vs. combined Q/S pathway contributions following CIH training).

One week post-CDR, ventral spinal BDNF and neurotrophin-3 concentrations are increased (Johnson et al., 2000), as is the strength of crossed spinal synaptic pathways to PMN (Fuller et al., 2002). Similarly, end-stage motor neuron disease (ALS) is associated with: (1) increased BDNF and other growth/trophic factor expression in PMN (Satriotomo et al., 2006); (2) increased serotonin terminal density; and (3) enhanced Q pathway dependent pLTF (Nichols and Mitchell, 2014). Thus, enhanced pLTF may occur via accentuation of the normal Q pathway to pLTF (i.e., following CDR and ALS), or additive contributions from the Q and S pathways to pMF (e.g., following CIH). Several proposed mechanisms for enhanced, AIH-induced pLTF are outlined within Figure 3.

## Significance

Although considerable effort has been devoted to investigations of detailed cellular mechanisms giving rise to AIH-induced pLTF (Devinney et al., 2013), relatively little attention has been given to the equally important concept of respiratory metaplasticity. We still lack a fundamental understanding of when, where and how respiratory metaplasticity occurs. Even though metaplasticity confers a remarkable potential to amplify (and harness) existing mechanisms of plasticity, metaplasticity also offers promise to reveal new forms of plasticity not present in normal animals. Examples of the latter include sensory LTF after CIH preconditioning (Peng and Prabhakar, 2003, 2004), and dAIH-revealed, AIH-induced hypoglossal LTF in Brown Norway rats; a strain that does not normally exhibit hypoglossal LTF (Wilkerson and Mitchell, 2009). While hypoglossal LTF and pLTF are phenotypically similar (i.e., enhanced nerve activity following prior experience), the threshold for eliciting each differs based on triggering stimulus and strain/species (reviewed in Golder et al., 2005), strongly suggesting an adaptive change in plasticity capacity/threshold following dAIH training.

With our significant progress in understanding cellular mechanisms of AIH-induced pLTF, it is an advantageous model to study metaplasticity in respiratory motor control. Such studies are warranted from a basic science perspective, but also because rAIH is rapidly moving towards clinical application as a treatment for respiratory insufficiency in disorders that compromise breathing capacity (Lovett-Barr et al., 2012; Nichols et al., 2013) as well as motor deficits in non-respiratory motor systems (Lovett-Barr et al., 2012; Hayes et al., 2014). Principles elucidated by studies of plasticity and metaplasticity in respiratory motor control may be an essential guide for understanding the plasticity, metaplasticity and functional recovery of a diverse range of clinical disorders that compromise movement (Lovett-Barr et al., 2012; Dale et al., 2014; Hayes et al., 2014).

## Conflict of interest statement

The authors declare that the research was conducted in the absence of any commercial or financial relationships that could be construed as a potential conflict of interest.
